# Supplementary material for: Association between SGLT2 Inhibitors and Cardiac Rehabilitation Outcomes in Patients with Cardiovascular Disease and Type 2 Diabetes Mellitus
Source: J Clin Med. 2022 Oct 9;11(19):5956. doi: 10.3390/jcm11195956 (PMC9571431; doi:10.3390/jcm11195956)
Supplement: Supplementary file 1 [file jcm-11-05956-s001.zip › jcm-1888427-supplementary.pdf]

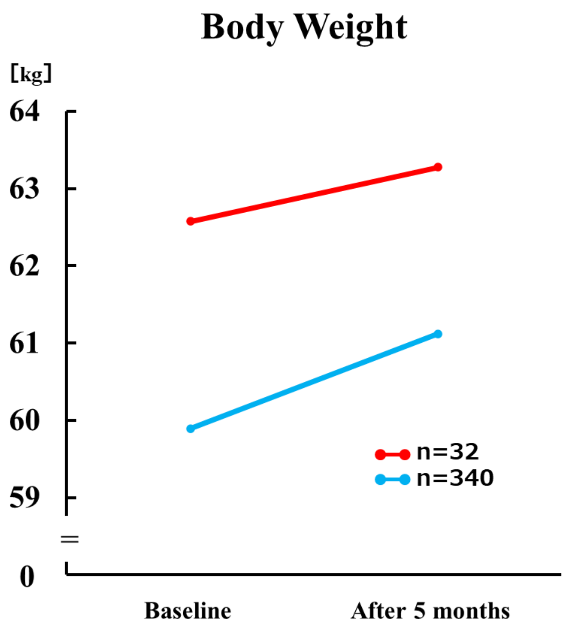

Supplemental Figure S1. Comparison of Δbody weight with and without SGLT2 inhibitor use in a mixed-effects model

Supplemental Table S1. Association of body weight with SGLT2 inhibitors use

Variables	Δ Body Weight	
	β	***P*-value**
SGLT2 inhibitors	0.434	0.070
Age	0.045	−0.211
Male	0.267	−0.114
BMI	0.481	0.132
NYHA class ≥ III	0.860	−0.014
LVEF	0.266	−0.103
HF	0.766	0.025
Hb	0.482	0.066
Year of hospitalization (≥ 2014)	0.331	−0.081
Baseline body weight	0.171	−0.302

SGLT2, Sodium-Glucose Cotransporter 2; BMI, body mass index; NYHA, New York Heart Association; LVEF, left ventricular ejection fraction; HF, heart failure; Hb, Hemoglobin
